# Urbanization increases fluctuating asymmetry and affects behavioral traits of a common grasshopper

**DOI:** 10.1002/ece3.9658

**Published:** 2022-12-21

**Authors:** Florian Rech, Nijat Narimanov, Tobias Bauer, Jens Schirmel

**Affiliations:** ^1^ iES Landau, Institute for Environmental Sciences University of Koblenz‐Landau Landau Germany; ^2^ Faculty of Environment and Natural Resources University of Freiburg Freiburg Germany; ^3^ Institute of Organismic and Molecular Evolution (iomE) Johannes Gutenberg University Mainz Germany; ^4^ State Museum of Natural History Karlsruhe Karlsruhe Germany

**Keywords:** activity, behavior, boldness, *Chorthippus biguttulus*, environmental stress, Orthoptera

## Abstract

Urbanization has a major impact on biodiversity. For many organisms, the urbanization process means environmental stress caused by fragmentation and increased temperatures in cities and atmospheric, soil, light, and noise pollution. Such environmental stress can influence both the morphology and behavior of animals. Hence, individuals might be selected for survival‐facilitating traits under high pressures in urban areas. The specific impact of urbanization on insect behavior is still largely unexplored. We studied the impact of urbanization on one of the most common grasshopper species in Germany, *Chorthippus biguttulus*, by comparing morphological and behavioral traits of individuals sampled from grasslands with low, medium, and high urbanization levels. We first investigated whether urbanization as a stressor affected body size and fluctuating asymmetry in the locomotor organs. Next, we examined whether urbanization induced changes in the individuals' boldness and activity. Our results showed that fluctuating asymmetry of grasshoppers' locomotory organs increased more than twofold with urbanization level. Further, individuals' boldness and walking activity increased from areas with low to high urbanization levels. Our results indicate strong responses of grasshoppers in terms of morphology and behavior to the urban environment. To compensate for urbanization effects on arthropod populations, management strategies need to be developed that maintain ecological processes and reduce environmental stress in urban areas.

## Graphical abstract

Urbanization increased fluctuating asymmetry of grasshoppers' locomotory. Individuals' boldness and walking activity increased with increasing level of urbanization. Our results indicate both detrimental (fluctuating asymmetry) and adaptive (higher mobility) responses of grasshoppers to the urban environment.

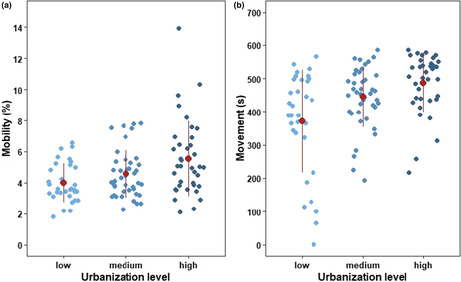

## 1 INTRODUCTION

Urbanization is a globally increasing phenomenon (Gaston, 2010) and one of the significant causes of land use change worldwide (Grimm et al., 2000). The accompanying increase in sealed impervious surfaces strongly alters local ecosystem conditions and material flows (Alberti, 2005; Hasan et al., 2020; Hutyra et al., 2014; McKinney, 2002; Tannier et al., 2016). A major concern related to urbanization are changes in biodiversity due to alterations in habitat connectivity (Beninde et al., 2015). Urbanization has been shown to negatively affect species richness and abundance for aquatic, limno‐terrestrial, and terrestrial invertebrates (Fattorini, 2011; Fenoglio et al., 2020; Piano et al., 2019; Schirmel, 2021) and is thus regarded as one of the greatest threats to global biodiversity (Seto et al., 2012). Besides its negative effects on many taxa, urbanization exposes species to environmental stress through environmental changes such as increased temperatures (urban heat island effect = heat accumulation in urban areas due to urban constructions), atmospheric and soil pollution, anthropogenic noise, and light pollution (Grimm et al., 2008). Conversely, urban areas can also contribute to the conservation of biodiversity and provide important habitats for birds of prey (Boal & Dykstra, 2018), insect pollinators (Derby Lewis et al., 2019; Hall et al., 2017), and arthropods (Buchholz et al., 2018).

Environmental stress can affect organisms in various ways, which include altered predation pressure, novel species interactions, and changes in food resource availability (Lowry et al., 2013). Moreover, environmental stress can increase phenotypic and genotypic variability of organisms (Hoffman & Parsons, 1991; Holloway et al., 1990; Pimpinelli & Piacentini, 2019). A widespread characteristic of this is the development of fluctuating asymmetry of bilateral traits. Fluctuating asymmetry is a measure of developmental stability, and the level of expression can be used as an indicator for different types of environmental stress (Clarke, 1995; Parsons, 1992). Fluctuating asymmetry has also been used in relation to urbanization for different animal groups such as mammals or insects (Beasley et al., 2013; Coda et al., 2017).

Further, environmental stress can induce intraspecific differences in physiological, ecological, and behavioral traits (Gunn et al., 2021; Gutiérrez et al., 2020; Kassahn et al., 2009; Koolhaas et al., 2011; Scherber et al., 2013). Thus, individuals from urban habitats may be characterized by physiological or behavioral traits that distinguish them from individuals inhabiting rural habitats. For example, individuals in anthropogenically modified environments are often flexible in behavior (phenotypic plasticity) (Lapiedra et al., 2016; Miranda et al., 2013) or show more bold and risk‐taking behavior toward enemies (Lowry et al., 2013; Schuett et al., 2010). Stress‐related behavior patterns prove to be good indicators of individual variation in behavior because they are applicable across species (Dingemanse et al., 2010). For example, individual differences in behavior can be very important in explaining variation in survival (Moiron et al., 2019). Typical behavioral patterns studied in animals include boldness and activity (Réale et al., 2007). Boldness describes an individual's response to a risky situation in a non‐novel environment, including reaction to predators and humans. Activity describes the general activity of an individual (Réale et al., 2007).

Selection may affect both sexes differently and can therefore cause sex‐specific differences in traits (Slatkin, 1984). Growing literature indicates that females and males differ in the mean levels of behavioral expression (Schuett et al., 2010) and repeatability of behavioral traits (Bell et al., 2009). Although a meta‐analysis has already shown that personality and traits are not sex‐dependent in most of the animals (Harrison et al., 2021), there may be sex‐specific differences with respect to certain traits in some species (e.g., sex‐specific dispersal, Han & Dingemanse, 2017; Li & Kokko, 2019; Trochet et al., 2016; exploration and aggression, Kralj‐Fišer et al., 2019, 2021; activity and risk‐taking behavior, Mezőfi et al., 2022), leading to the evolution of sex‐specific genetic architecture behind such traits (Lande, 1980). In addition, responses to environmental stress may be sex‐dependent. For example, males and females of the butterfly *Melitaea cinxia* responded differently to larval food stress and warm‐night temperatures (Rosa & Saastamoinen, 2017, 2021) and the nonbiting midge *Chironomus riparius* showed sex‐specific responses to pesticide exposition (Roodt et al., 2022).

Behavioral differences between rural and urban individuals of a species are widespread across bird species, underscoring a substantial ecological influence of urbanization on animal behavior (Miranda et al., 2013). Urbanization has also been shown to be a stress to insects, including grasshoppers. For instance, urban individuals of the grasshopper *Chorthippus brunneus* showed higher levels of mobility‐related morphology traits than those from the rural populations (San Martin y Gomez & Van Dyck, 2011). However, the empirical evidence of the impact of urbanization on insect morphological and behavioral traits is still very limited (Diamond et al., 2015).

Here, we studied the impact of urbanization on morphological (fluctuating asymmetries and length of the femora and tegmina) and behavioral traits (boldness, activity) of one of the most common European grasshopper species, *Chorthippus biguttulus*. We sampled individuals in the field from grassland sites differing in their urbanization levels (low, medium, and high) and investigated fluctuating asymmetry of the forewings and the femora, boldness, and activity patterns. We hypothesized that (i) urbanization increases the expression of fluctuating asymmetry in locomotor organs of *C. biguttulus* and that individuals inhabiting more built‐up areas are (ii) bolder and (iii) more active than counterparts from the less urbanized areas. We further investigated whether the effect of urbanization on traits was sex‐dependent.

## 2 MATERIALS AND METHODS

### 2.1 Study species and sampling sites

*Chorthippus biguttulus* is one of the most common grasshoppers in Central Europe (Bellmann et al., 2019). In Germany, *C. biguttulus* is widely distributed in various grassland habitats with a preference for dry‐warm conditions and sandy substrates (Detzel, 1998). The species is herbivorous and feeds on leaves of different plant families (Zahid et al., 2017).

Grasshoppers were collected from 15 sites in the greater Karlsruhe area (Baden‐Wuerttemberg, Germany; Appendix S1). The sites consisted of extensively managed meadows in urban green spaces that serve as suitable habitats for the target species. Sampling sites were selected according to their surrounding urbanization level. For this purpose, a buffer of a 500 m radius was set around each site, and the total amount of buildings, residential and industrial area was determined using digital land‐use maps from OpenStreetMaps (OSM), supplemented with land‐use maps of green spaces provided by the greenspace administration of Karlsruhe (Gartenbauamt Karlsruhe) using QGIS Desktop 2.18.3 (QGIS Development Team, 2017). Public parks, graveyards and greenspaces, meadows, cropland, forest‐ and scrubland, railroads and allotments were not considered as urbanized area.

The sites were then classified into three categories: “low” with a proportion of <10%, “medium” with 30%–50%, and “high” with >60% of urbanization in the 500 m buffer. For each of the categories, five sites were sampled. Sampling with a sweep net was conducted in August 2020 for a total of 6 days during dry weather conditions. When walking the sample sites the sweep net was swung twice per step and examined for individuals of *C. biguttulus*. We aimed to sample 10 individuals per site, and finally, 149 individuals (92 females and 57 males) were collected for the experiments.

### 2.2 Rearing of the individuals in the laboratory

After sampling in the field, individuals were individually caged in round microcosms (radius: 5.9 cm, volume: 870 ml) in Landau, Germany. Each animal received an unique encrypted code to make the sample area and its degree of urbanization unrecognizable during the experiments to avoid experimenter bias (Martin & Bateson, 1993). The microcosms contained sand as a soil layer and were stored in a climate‐controlled chamber under standard conditions (day = 25°C, night = 20°C, RH = ~65%, L:D = 15:9) to provide optimal living conditions for grasshoppers. The animals were fed fresh grass (Poaceae) every 2 days. To simulate near‐natural humidity conditions like dew and precipitation, each microcosm was humidified every 2 days with three sprays of water. Each animal was kept under the conditions described above for at least 24 h before being prepared for the first experiment. Between experimental runs, the caging time was also at least 24 h. After the experiments, the animals were stored in 70% 1,2‐propanediol until morphological measurements were taken to determine fluctuating asymmetries.

### 2.3 Fluctuating asymmetry

Two different metric parameters were examined and measured for fluctuating asymmetry in the target species. Because paired traits that are as large as possible are needed for the statistical evaluation of fluctuating asymmetry, the length of the forewings (tegmina), and the femora of the jumping legs were measured (Van Dongen et al., 2008). All measurements were made by the same person using a digital electronic caliper (Preciva) with a measurement accuracy of 0.01 mm, under a stereo microscope. The measurements of femora and tegmina were conducted three times and the mean was used to determine the asymmetries. In preparation for the measurements, the hind legs were removed from the body using forceps. Then the femora were separated from the adjacent tibia and coxa. The femora were attached to white cardboard with transparent adhesive tape to avoid slipping of the body parts during the measurement procedure. The most distant points of the outer edge of the upper, larger lobe of the notched base, and the outer edge of the upper of the two genicular lobes at the apex were chosen as measurement points of the femur. In preparation for the measurements of the tegmina, first, the entire wing apparatus under the pronotum was removed. Subsequently, the forewings were separated. For the measurement, the forewings were smoothed with a microscope slide and then attached to white cardboard using transparent adhesive tape. For the measurements of the tegmina, the measurement points were the starting point of the marginal membrane at the base of the forewing and the rounded apex of the forewing.

### 2.4 Boldness

In risky situations, such as the appearance of predators or humans, the expression of boldness in grasshoppers can be observed (Niemelä et al., 2011; Nishino & Sakai, 1996). Because birds and other animals often grab grasshoppers, they defend themselves by using tonic immobility and this defensive response to a predator can be interpreted as boldness (Ruxton, 2006). This tonic immobility can be simulated and used to interpret the boldness or risk‐taking of individual grasshoppers. Hence, to measure boldness of the animals, the tonic immobility of each individual was quantified. The animal was grabbed by both jumping legs at the tibia and held in the air about 1.50 m above the ground. After an acclimation period of 10 s, the time was stopped within 30 s to see how long the grasshopper remained in a rigid position. The shorter the individual used tonic immobility, the more pronounced its boldness. The experiment was performed three times on each individual (with pauses of 5 min between trials) to evaluate the mean and reduce outlier values and extreme deviations.

### 2.5 Activity

For the measurement of activity, an open field test was performed (Appendix S2). This test is generally used to observe the activity and exploration of animals (Réale et al., 2007). The basic assumption in performing the test is that the animals used are exposed to an unknown open area that offers them no possibility of retreat. Thus, the activity of the animals can be observed, which depends on various factors such as fear behavior, readiness to flee, and exploration. For the open field test, a suitable arena was prepared. A transparent Plexiglas foil was cut to size and glued together in a cylindrical shape (diameter: 50 cm). The grasshoppers have a pronounced hiding behavior and seek retreats in danger (Hassenstein & Rustert, 1999). The arena, hence, was designed round in order not to offer the animals shelters in corners that could influence their behavior. The floor of the arena was fitted with white cardboard to provide better video footage for analysis. The arena was covered with a glass plate to prevent the animals from escaping. Outside the arena, a tripod with a video camera (Panasonic 4 K Ultra HD‐Camcorder HC‐VXF990; Panasonic Corporation) was set up to record the activity of the animals (Appendix S3). The video camera was placed at a 180° angle to film the entire arena from above. Each animal was filmed for a total of 10 min and 20 s. The recording was started, then the animal was deployed in the center of the arena, and the glass plate was placed on top of the arena as a lid. During the trials, we left the room to reduce the stress factor (Mestre et al., 2020). For the analysis of activity, the first 20 s were discarded so that exactly 10 min of video footage was analyzed for each animal.

To analyze the activity, we used EthoVision XT12 (Noldus Information Technology), which allows the automation of behavioral observations (Noldus et al., 2001). The parameters studied were distance traveled, mobility, and movement. Distance traveled describes the total distance traveled from the center of the marked animal body in 10 min. Mobility describes the percentage of changed pixels of the detected object on average. Mobility is defined as the degree of movement of the animal's body independent of the spatial displacement of the center of the body (Noldus et al., 2001). Therefore, note that an animal may have high mobility and yet, at the same time, a low movement. Measurement of mobility is often used to demonstrate inferences about immobility in fear‐related behavior (Pham et al., 2009). Movement determines the periods of time during which the center of the marked body of the animal is in motion or not in motion. The status is characterized by the values “in motion” and “not in motion”. In order to measure these values correctly and avoid measurement errors, measurement limits were defined. The status “in motion” is applied from the time when the average speed of the center of the marked body exceeds a defined starting speed. The state “not in motion” occurred as soon as the average velocity of the marked body center fell below a defined stop velocity. The measurement limit selected for the start velocity was 0.1 cm/s and for the stop velocity was 0.05 cm/s.

### 2.6 Statistical analysis

In total, 149 (92 females and 57 males) individuals were used for the measurements of fluctuating asymmetry, 132 (83 females and 49 males) individuals were tested for boldness behavior, and 111 individuals (67 females, 44 males) for activity. The number of individuals used for the different experiments decreased because some grasshoppers died over the rearing period.

To test the correlation between fluctuating asymmetries, Spearman‐correlation was applied. For all data, linear mixed‐effects models were created in R 4.0.5 (R Core Team, 2021) using the command “lmer” from the R package “lme4” (Bates et al., 2015). The urbanization level (factor with three levels: low, medium, and high), grasshoppers' sex (factor with two levels: male and female) and interaction of both (urbanization level × sex) were included in the models as fixed factors. To account for the presence of multiple individuals collected from the same sample plots, plot IDs (factor with 15 levels) were included as random effects in the models. Subsequently, the significance of the explanatory variables was calculated using type‐II analysis‐of‐variance‐tables based on Wald Chi^2^ tests using the “ANOVA” command from the R package “car” (Fox & Weisberg, 2019).

## 3 RESULTS

### 3.1 Fluctuating asymmetry

**FIGURE 1:**
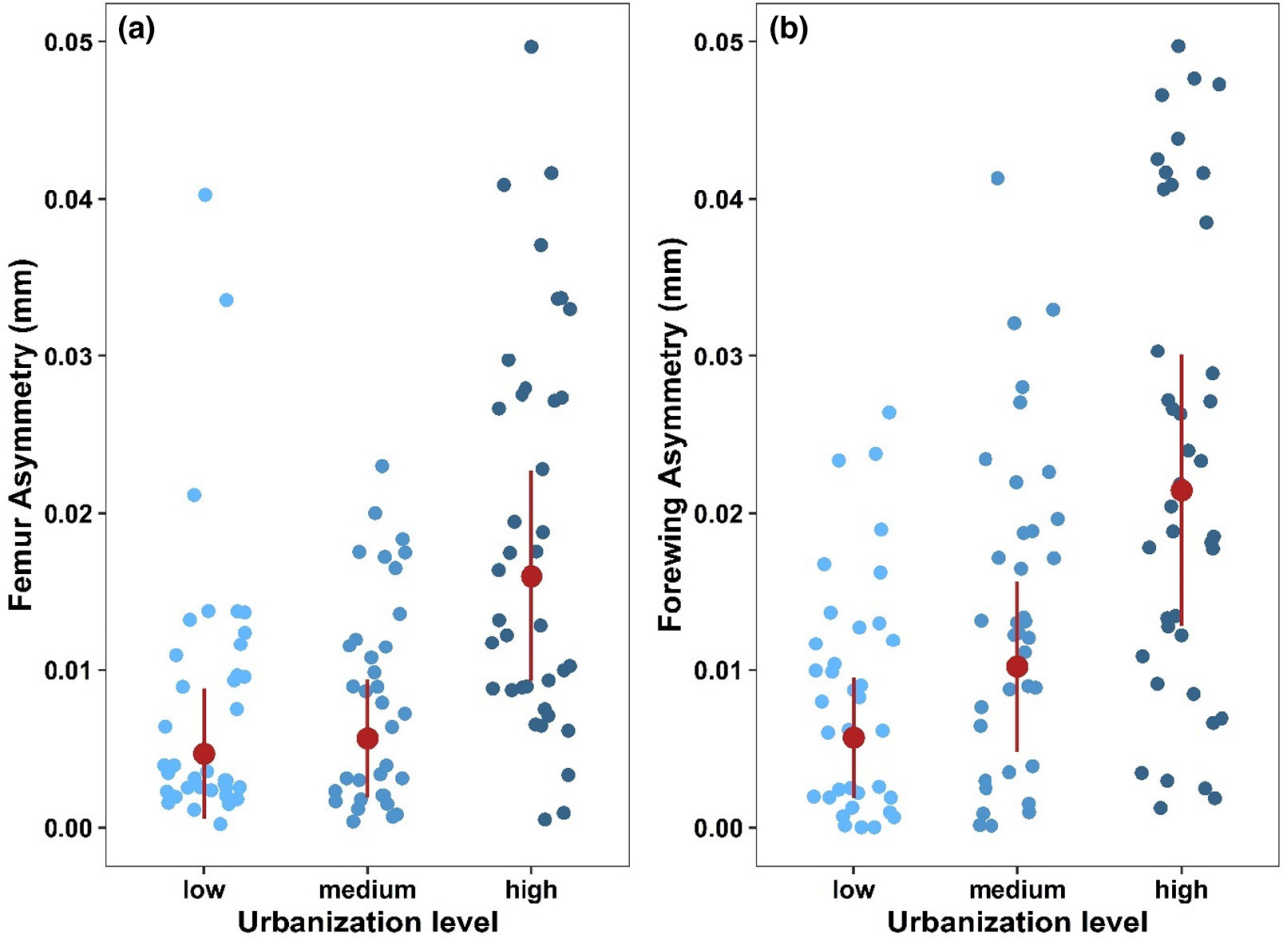
Effects of urbanization (low, medium, and high) on fluctuating asymmetry (mm) in (a) the femora and (b) the tegmina of *Chorthippus biguttulus*. Plots include raw data points, mean, and standard deviation.

Urbanization had no impact on femur and tegmina length (Table 1). With increasing levels of urbanization, fluctuating asymmetries occurred significantly more frequently in both the tegmina and femora of *Chorthippus biguttulus*. Thereby, individuals showed an increase of 242% for the tegmina and 276% for the femora fluctuating asymmetry from the lowest to the highest urbanization level (Figure 1a,b). The fluctuating asymmetries correlated positively with each other (Spearman‐*r* = .51). Sex and the interaction between urbanization and sex did not significantly affect the fluctuating asymmetry of tegmina and femora (Table 1).

**TABLE 1:** Results of linear mixed effect models testing the relations of morphological and behavioral traits of *Chorthippus biguttulus* to urbanization, sex, and their interaction (fixed effects).

Trait variable	Explaining variables	χ^2^	*p*
Femur length	Urbanization	3.710	.156
	Sex	1411.65	**<.001**
	Urbanization × Sex	0.457	.796
Tegmina length	Urbanization	4.254	.1192
	Sex	886.013	**<.001**
	Urbanization × Sex	1.913	.3842
Fluctuating asymmetry tegmina	Urbanization	38.872	**<.001**
	Sex	3.195	.074
	Urbanization × Sex	4.871	.088
Fluctuating asymmetry femur	Urbanization	34.964	**<.001**
	Sex	0.011	.917
	Urbanization × Sex	0.547	.761
Boldness	Urbanization	13.407	**.001**
	Sex	0.002	.967
	Urbanization × Sex	0.424	.809
Traveled distance	Urbanization	2.940	.223
	Sex	0.807	.369
	Urbanization × Sex	1.105	.576
Mobility	Urbanization	10.079	**.007**
	Sex	0.010	.919
	Urbanization × Sex	0.590	.745
Movement	Urbanization	17.821	**<.001**
	Sex	2.995	.084
	Urbanization × Sex	1.928	.381

*Note*: To account for the presence of multiple individuals collected from the same sample plots, plot IDs were included as random effects in the models. The significance of the explanatory variables was calculated based on Chi^2^ statistics, and significant results (*p* < .05) are shown in bold.

### 3.2 Boldness

**FIGURE 2:**
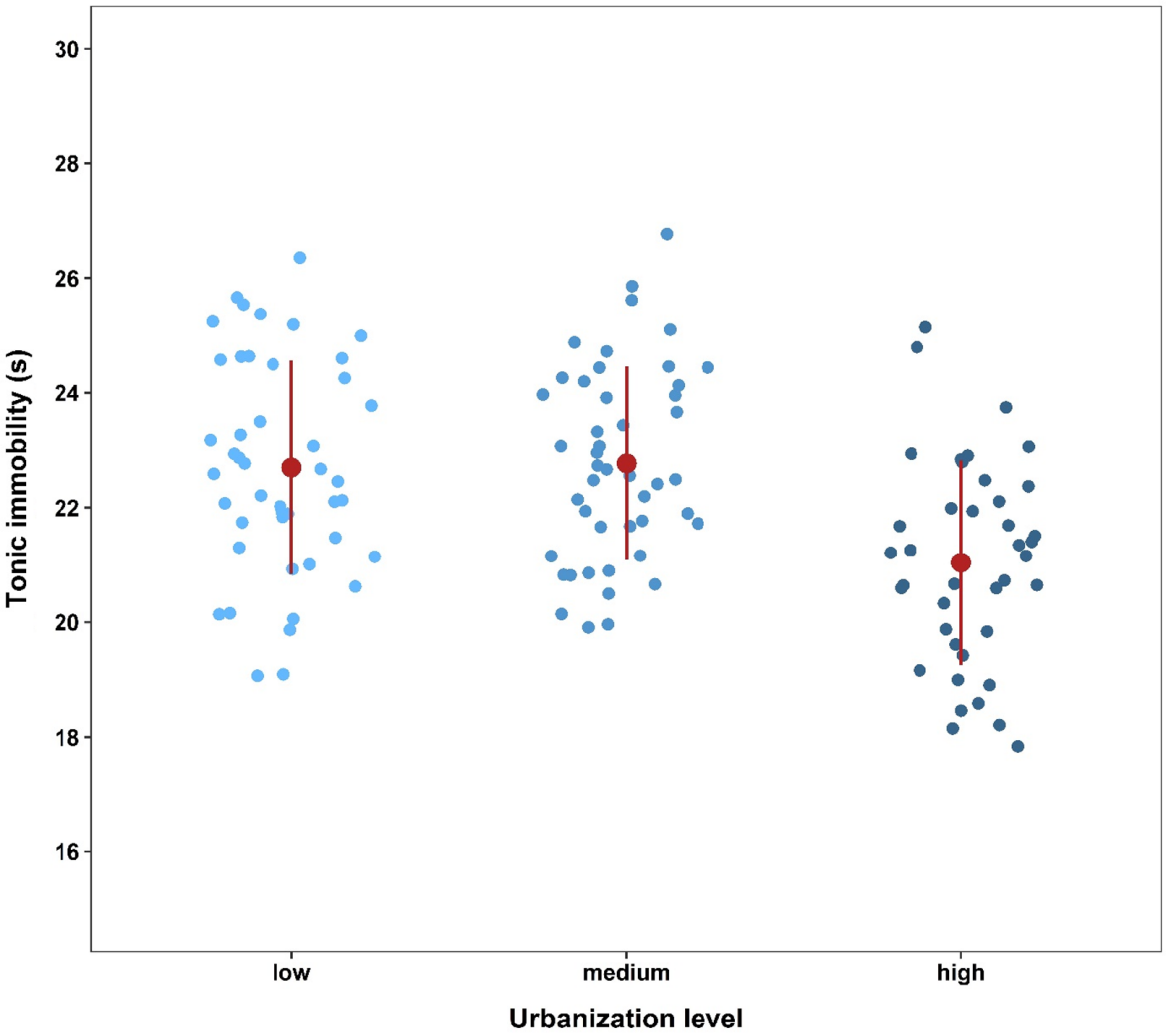
Effects of urbanization (low, medium, and high) on boldness (tonic immobility; s) of *Chorthippus biguttulus*. Plots include raw data points, mean, and standard deviation.

Boldness of individuals significantly increased with the level of urbanization (Table 1; Figure 2). Individuals from highly urbanized sites showed, on average, a 12% shorter tonic immobility than animals from sites with a low degree of urbanization. Both female and male individuals exhibited a similar level of boldness irrespective of urbanization level (urbanization level × sex; Table 1).

### 3.3 Activity

**FIGURE 3:**
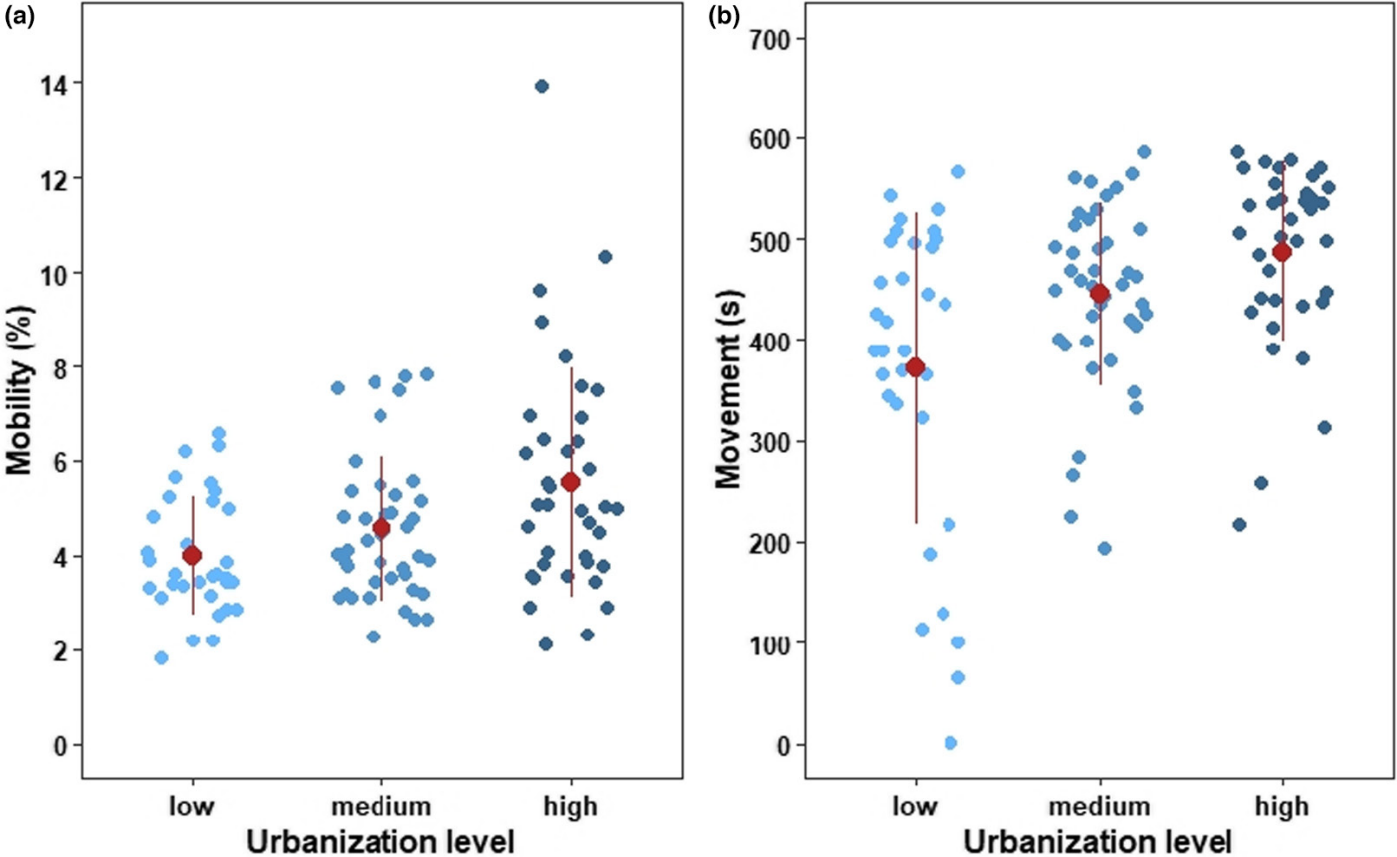
Effects of urbanization (low, medium, and high) on (a) mobility and (b) movement of *Chorthippus biguttulus*. Plots include raw data points, mean, and standard deviation.

Grasshoppers' mobility and movement significantly increased with urbanization level. Individuals from areas with high urbanization were 30% more mobile and had a longer movement time than counterparts from areas with medium and low urbanization levels (Table 1, Figure 3a,b). Individuals from areas with high, medium, and low levels of urbanization moved similar distances during our experiments (Table 1). Further, both sexes showed similar rates of all activity parameters irrespective of urbanization levels (urbanization level × sex; Table 1).

## 4 DISCUSSION

Our results indicate that urban individuals experienced higher environmental stress throughout their development, expressed by the higher levels of asymmetry. As hypothesized, the fluctuating asymmetry in the studied body parts, namely tegmina and femora, of *Chorthippus biguttulus* steadily increased with an increasing degree of urbanization. Both fluctuating asymmetries are strongly correlated, which indicates that when an individual exhibits fluctuating asymmetry, it occurs in both body parts. Fluctuating asymmetry is a reliable biomarker of environmental stress impacting individuals (Beasley et al., 2013; Leung et al., 2000), which has previously been shown for other stressors besides urbanization, such as, for example, agrochemical pollution (Jentzsch et al., 2003), natural disasters (Gerard et al., 2018), and parasitism (Uetz et al., 2009). However, it is challenging to answer which urban stress factor is crucial in affecting fluctuating asymmetry. Presumable stressors would be artificial light at night, noise pollution, a higher number of stressful encounters with people (and their dogs), increased experience of local drought periods, temperature changes, or chemical pollution (Beasley et al., 2013). The effects of urbanization on *C. biguttulus* can affect the performance of individuals in several ways. Femora have a stronger function than tegmina for the movement pattern of *C. biguttulus* since individuals of this species move more by running and jumping than by flying; moreover, the tegmina only play a minor role in active flying and are more important for song production since the hindwings are the main flying organs in grasshoppers (Detzel, 1998). We observed a slightly less pronounced fluctuating asymmetry with increasing urbanization in the femora than in the tegmina. This supports the assumption that body parts whose symmetry is more important for certain functions are more resistant and less susceptible to fluctuating asymmetry. Body parts that do not strongly influence the individual fitness of the target organism, such as tegmina in *C. biguttulus*, have a lower buffering capacity for fluctuating asymmetry and tend to develop fluctuating asymmetry more strongly (Clarke, 1995; Markow, 1995). Asymmetry in the locomotor extremities (here mainly the femora) is considered detrimental because it causes locomotor limitations (Møller, 1993). For this reason, among others, the presence of fluctuating asymmetry might negatively affect the individual fitness of animals (Beasley et al., 2013). For *C. biguttulus* and grasshoppers in general, restricted movement means restricted escape behavior from predators and an increased risk of being preyed upon.

We tested the boldness behavior of *C. biguttulus* in the form of a simulated attack situation. This evoked tonic immobility in the animals, representing a defensive response to predation and thus can be interpreted as bold behavior. Thereby, boldness is a behavioral pattern that is often related to the individual activity (Réale et al., 2007). As hypothesized, we found that *C. biguttulus* individuals from highly urbanized habitats were bolder (= lower tonic immobility) and thus expressed more risk‐taking behavior than individuals from less urbanized habitats. This is an often‐observed behavioral pattern in many animal species colonizing urban habitats including birds (Atwel et al., 2012; Scales et al., 2011), lizards (Baxter‐Gilbert et al., 2019), and insects, as has been shown for example for carabid beetles (Magura et al., 2021; Schuett et al., 2018). A meta‐analysis of different animal groups found that, on average, bolder individuals have higher reproductive success and show versatile trade‐offs in the factors of survival and fitness (Smith & Blumstein, 2008). However, it remains to be investigated whether lower tonic immobility of *C. biguttulus* provides advantages in a natural predator–prey situation, and thus whether individuals from more urbanized habitats have higher survival and a fitness advantage over individuals from less urbanized habitats.

In support of our hypothesis, *C. biguttulus* individuals from highly urbanized habitats were more active in terms of mobility (i.e., degree of movement of the animal's body independent of the spatial displacement) and movement (i.e., time of the animal in motion) than those from less urbanized habitats. We used an open field test, where animals were exposed to an unknown open area that offered them no possibility of retreat. The observed activity patterns could therefore be related to the exploration behavior of animals, including fear behavior and readiness to flee (Réale et al., 2007). One of the main factors determining an animal's activity is the amount of time it spends moving (Noldus et al., 2001). The time spent in movement *by C. biguttulus* was found to be increased by one quarter for individuals from highly urbanized compared to those of lowly urbanized habitats. Mobility is regarded as an important factor in escape behavior. It can be assumed that animals that show higher general mobility have higher chances of a successful escape attempt in risky predator–prey situations. In general, grasshoppers tend to avoid risky situations by hiding and showing distinct escape behaviors (Ingrisch & Köhler, 1998). The success of the latter depends on the speed of the individuals and the mobility of their extremities (Hassenstein & Rustert, 1999). The observed higher mobility of individuals from highly urbanized habitats might therefore be an adaption to high predation pressure in cities and evidence for trait variability caused by urbanization (Alberti, 2014; Bolnick et al., 2011). However, activity is a behavior in which fitness tradeoffs are made (Réale et al., 2007). Higher activity of individuals positively affects fitness because it allows for better escape behavior. At the same time, increasing activity enhances the probability of risky situations. Therefore, it should be investigated whether a higher activity of individuals in urban areas indeed provides a fitness advantage.

Although sex may influence the expression of traits, our results on *C. biguttulus* show that boldness and activity are sex independent in this common grasshopper. Further, no differences in asymmetry could be observed between the sexes. Intersexual differences would suggest that particular sex responds more or less strongly to the stressor of urbanization. Also, a difference in boldness behavior between sexes was not observed, indicating that boldness is a sex‐independent behavioral pattern in *C. biguttulus*. Although different sexes may exhibit different activity patterns in grasshopper species (Forsman & Appelqvist, 1999), these could not be detected in *C. biguttulus*.

## 5 CONCLUSION

Our study shows that urbanization acts as environmental stress, affecting fluctuating asymmetry in locomotor organs in a common grasshopper species. Furthermore, selection for individuals with higher activity rates and boldness in highly urbanized areas indicates the fitness‐facilitating character of these behavioral traits in *C. biguttulus*. In the wake of current global change, management strategies need to be intensified to mitigate increasing environmental stress in urban areas and hence contribute to better conservation strategies for urban arthropod communities worldwide.

## AUTHOR CONTRIBUTIONS

**Florian Rech:** Conceptualization (lead); data curation (lead); formal analysis (equal); investigation (lead); methodology (equal); project administration (lead); resources (equal); software (lead); validation (equal); visualization (lead); writing – original draft (lead); writing – review and editing (equal). **Nijat Narimanov:** Conceptualization (supporting); data curation (supporting); formal analysis (equal); investigation (supporting); methodology (equal); project administration (supporting); resources (equal); software (lead); supervision (supporting); validation (equal); visualization (lead); writing – original draft (supporting); writing – review and editing (equal). **Tobias Bauer:** Conceptualization (supporting); formal analysis (equal); investigation (supporting); methodology (equal); supervision (supporting); validation (supporting); writing – original draft (supporting); writing – review and editing (equal). **Jens Schirmel:** Conceptualization (lead); data curation (supporting); formal analysis (equal); investigation (supporting); methodology (supporting); project administration (supporting); resources (equal); software (supporting); supervision (lead); validation (equal); visualization (supporting); writing – review and editing (equal).

## CONFLICT OF INTEREST

The authors declare no conflict of interest.

## Supporting information


**Appendix S1.** Sampling sites with associated coordinates and degree of sealed surfaces surrounding the sampling sites within a 500 m buffer. Sampling sites are classified into three categories of urbanization based on the degree of sealing (L = low, M = medium, and H = high).

**Appendix S2.** Graphical scheme of the experimental setup for tracking the activity of individuals. Each individual was placed in the center of the arena and recorded for 10 minutes.

**Appendix S3.** Videoclip of the activity measurements.

## Data Availability

Generated and analyzed data during this study are available from Figshare: https://doi.org/10.6084/m9.figshare.19944374.
