# Supplementary material for: Urbanization increases fluctuating asymmetry and affects behavioral traits of a common grasshopper
Source: Ecol Evol. 2022 Dec 21;12(12):e9658. doi: 10.1002/ece3.9658 (PMC9772494; doi:10.1002/ece3.9658)
Supplement: Supplementary file 1 — Appendix S1. Sampling sites with associated coordinates and degree of sealed surfaces surrounding the sampling sites within a 500 m buffer. Sampling sites are classified into three categories of urbanization based on the degree of sealing (L = low, M = medium, and H = high). [file ECE3-12-e9658-s002.docx]

**Appendix S1**

Sampling sites with associated coordinates and degree of sealed surfaces surrounding the sampling sites within a 500 m buffer. Sampling sites are classified into three categories of urbanization based on the degree of sealing (L= low, M=medium, H=high).

| **Site** | **Coordinates** | **Sealing** |
| --- | --- | --- |
| L1 | 48.992736, 8.317180 | < 0.1 |
| L2 | 49.041097, 8.460295 | < 0.1 |
| L3 | 48.976901, 8.490774 | < 0.1 |
| L4 | 48.979923, 8.444185 | < 0.1 |
| L5 | 48.972115, 8.325152 | < 0.1 |
| M1 | 48.996449, 8.375054 | 0.45 |
| M2 | 48.981232, 8.400559 | 0.44 |
| M3 | 49.037695, 8.386000 | 0.37 |
| M4 | 49.023572, 8.448358 | 0.47 |
| M5 | 48.990119, 8.390247 | 0.48 |
| H1 | 49.034661, 8.443086 | 0.66 |
| H2 | 49.007030, 8.353190 | 0.65 |
| H3 | 49.004584, 8.344039 | 0.63 |
| H4 | 49.032625, 8.364318 | 0.66 |
| H5 | 49.003313, 8.348767 | 0.65 |
