# Supplementary material for: Urbanization increases fluctuating asymmetry and affects behavioral traits of a common grasshopper
Source: Ecol Evol. 2022 Dec 21;12(12):e9658. doi: 10.1002/ece3.9658 (PMC9772494; doi:10.1002/ece3.9658)
Supplement: Supplementary file 2 — Appendix S2. Graphical scheme of the experimental setup for tracking the activity of individuals. Each individual was placed in the center of the arena and recorded for 10 minutes. [file ECE3-12-e9658-s001.docx]

**Appendix S2**

Graphical scheme of the experimental setup for tracking the activity of individuals. Each individual was placed in the center of the arena and recorded for 10 minutes.

**
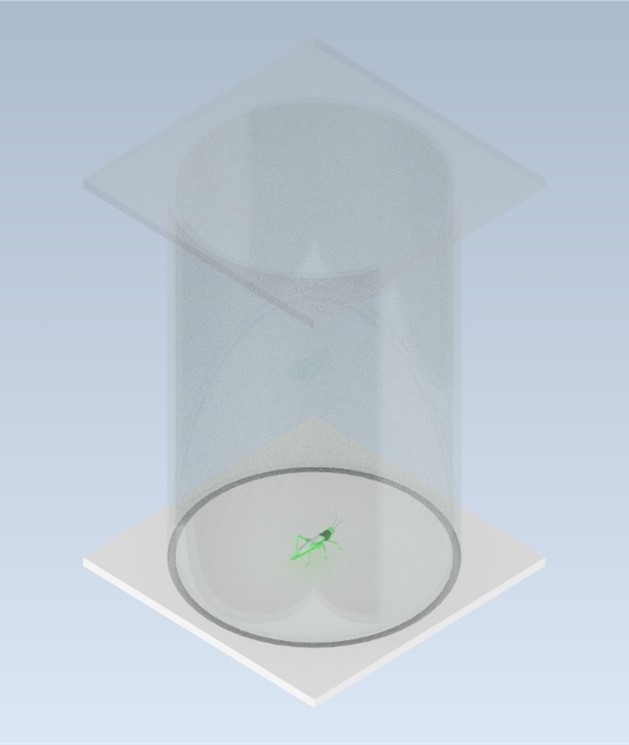
**
